# Effect of Lighter and Heavier Initial Weight on Growth Performance and Carcass Traits of Single-Source Beef Steers

**DOI:** 10.3390/ani14040567

**Published:** 2024-02-08

**Authors:** Thomas C. Norman, Erin R. DeHaan, Forest L. Francis, Warren C. Rusche, Zachary K. Smith

**Affiliations:** Department of Animal Science, South Dakota State University, Brookings, SD 57007, USA; thomas.norman@sdstate.edu (T.C.N.); erin.gubbels@sdstate.edu (E.R.D.); forest.francis@sdstate.edu (F.L.F.); warren.rusche@sdstate.edu (W.C.R.)

**Keywords:** beef, body weight, dietary net energy

## Abstract

**Simple Summary:**

This study aimed to determine the influence that initial BW has on growth performance responses, efficiency of dietary net energy (NE) utilization, and carcass traits in feedlot steers. Experimental data were analyzed as a randomized complete block design with pen as the experimental unit. Heavy initial weight (HIW) steers had greater growth, but poorer feed efficiency compared to light initial weight (LIW) steers. Steers with HIW produced fatter carcasses with a greater degree of marbling.

**Abstract:**

The objective of the study was to determine the influence that initial BW has on growth performance responses, efficiency of dietary net energy (NE) utilization, and carcass traits in feedlot steers. Charolais×Red Angus steers (*n* = 70) selected from a larger single-source group were used in a 209-d growing-finishing feedlot experiment. Steers were assigned to two groups based on initial BW (light initial weight, LIW = 273 ± 16.0 kg; heavy initial weight, HIW = 356 ± 14.2 kg) and allotted into 10 pens (*n* = 7 steers per pen; 5 pens per experimental group) the within pen standard deviation for LIW was from 14.1 kg to 20.9 kg and for HIW was from 13.7 kg to 16.0 kg. Steers were fed a common diet once daily. Experimental data were analyzed as a randomized complete block design with pen as the experimental unit. LIW steers had a greater cumulative HH change (*p* = 0.04). A treatment × day interaction (*p* = 0.05) was observed for HH with HIW steers having a greater HH at all time points. Final BW and carcass-adjusted (HCW/0.625) BW were greater for HIW steers by 13.1% and 13.4% respectively (*p* ≤ 0.01). HIW steers had a greater DMI (*p* = 0.01) compared to LIW. Cumulative ADG was greater for HIW by 3% (*p* = 0.04). LIW steers had better feed conversion (*p* = 0.01). HIW steers had greater (*p* ≤ 0.05) HCW, marbling scores, and yield grade (YG), with decreased REA/HCW (*p =* 0.01) compared to LIW. The distribution of USDA Yield Grade was altered by initial BW (*p* = 0.04). No differences were detected (*p* ≥ 0.22) for the distribution of Quality grade nor liver abscess prevalence and severity. Regression coefficients did not differ between LIW and HIW for urea space calculations of empty body water, fat, or protein (*p* ≥ 0.70). A quadratic response was noted for empty body fat (EBF), empty body water (EBH20), and carcass protein (CP). In conclusion, HIW steers had greater growth, but poorer feed efficiency compared to LIW steers. Steers with a HIW produced fatter carcasses with a greater degree of marbling.

## 1. Introduction

Cattle feeders in the Northern Plains of the United States routinely feed two distinct diets to livestock during production. Forage-based diets (1.05 to 1.19 Mcal/kg NEg) are commonly fed during the backgrounding phase with concentrate-based diets (1.32 to 1.50 Mcal/kg NEg) fed during the finishing phase. Overall goals of backgrounding programs include: (1) managing disease and health, (2) achieving economical gains, (3) enhancing finishing phase feed conversion, (4) optimizing total carcass weight gain, and (5) managing feeder cattle supply into the feedlot phase of production. Previous work within our lab group conducted by Hamilton [1] demonstrates cattle fed a single growing-finishing diet had similar growth performance and carcass traits upon harvest at equal days on feed as compared to steers fed within a two-diet phase system. However, in that experiment we used steers that were uniform in weight. To the best of our knowledge, only one other study evaluated the influence of initial weight grouping on productive responses using calf-fed Holstein steers reared in confinement [2]. Determining the frame score requires hip height and age data; these are two factors that are typically unknown at the feedlot, while frame score equations were developed from cattle data generated in the 1970s and are unlikely to correctly describe modern cattle phenotypes [3]. Close-out analysis of feedlot cattle using data from commercial feedlots indicated that as the initial weight at placement increased, daily gain, intake and final weight increased, while the feed efficiency was reduced; however, this large pen close-out analysis did not include findings regarding carcass trait responses [4]. To account for differences in mature size, it was initially proposed to use the equivalent body weight system to estimate energy requirements for gain and protein deposition [5,6]. The use of the equivalent BW system was ultimately adopted in the NASEM updates in 1996 and again in 2016 [7,8]. An evaluation of the feeder cattle frame size and carcass outcomes indicated that previous standards were confounded by body condition and muscularity [9]. Calves with similar initial BWs but divergent genotypes fed a high-concentrate diet had differing carcass outcomes, and these findings indicate that genetics played a larger role in carcass outcomes compared to the initial placement BW [10]. It was not clear if using steers with similar genetics and differing initial weights would demonstrate the same growth responses when fed a single diet for an extended growing-finishing period. The objective of this experiment was to determine the influence that body weight at the initiation of the growing-finishing phase had on growth performance responses, changes in body composition, efficiency of dietary net energy (NE) utilization, and carcass traits in steers fed a single diet during a 209-d growing—finishing period.

## 2. Materials and Methods

### 2.1. Use of Animal Subjects

This study was conducted at the Ruminant Nutrition Center (RNC) in Brookings, SD, USA between December 2021 and July 2022. Animal care and handling procedures used in this study were approved by the South Dakota State University Institutional Animal Care and Use Committee (Approval Number: 2110-063A).

### 2.2. Animals, Initial Processing, and Study Initiation

Pre-conditioned crossbred beef steers (*n* = 70; initial shrunk [4%] BW = 329 ± 72.6 kg) were used in a 209-d experiment at the Ruminant Nutrition Center (RNC) in Brookings, SD. Steers were fed once daily, and bunks were managed according to a slick bunk management system. Light- and heavy-weight Charolais × Red Angus steers selected from the heavy and lighter tails of a larger group from a single South Dakota ranch were used in this experiment. Steers received approximately 2 months (56 d) before this study’s initiation were used. Cattle were fed in 7.62 × 7.62 m concrete surface pens (*n* = 10 pens total; 7 steers/pen; 5 replicate pens/treatment mean) with 7.62 m of bunk space and heated, concrete, continuous flow waterers.

Steers were vaccinated at receiving against viral respiratory diseases (Bovishield Gold 5; Zoetis, Parsippany, NJ, USA), clostridial species (Ultrabac 7/Somubac, Zoetis), and administered pour-on moxidectin (Cydectin, Bayer, Shawnee Mission, KS, USA) before the initiation of this study. At study initiation, steers were weighed (scale readability 0.454 kg) and processed on d − 1 and were weighed again and allocated to study pens on d 1. Individual BW and hip heights (HH) were collected at study initiation. Steers were administered an implant on d 1 (100 mg trenbolone acetate and 14 mg estradiol benzoate; Synovex Choice, Zoetis) and reimplanted on d 112 (200 mg trenbolone acetate and 28 mg estradiol benzoate; Synovex Plus, Zoetis). Implant retention was checked on d 63 and d 153 by a trained technician with no major abnormalities (i.e., abscessed, missing, or partial) noted [11].

### 2.3. Experimental Design and Treatments

This experiment was a randomized complete block design with 7 steers per pen, blocked by batch fraction fed [12]. A total of 10 pens were used with 5 replicates per experimental group. Treatments included (1) Light Initial Weight (LIW) and (2) Heavy Initial Weight (HIW).

### 2.4. Dietary Management

Steers were fed a common diet containing 16% roughage (13.1% CP and 23.4% NDF; Table 1). Finishing diets consisted of dry rolled corn (DRC), high moisture corn (HMC), liquid supplement (LS), dried distiller’s grains (DDGS), and corn silage (CRNSIL). The LS provided monensin sodium (Rumensin 90; Elanco, Indianapolis, IN, USA) at 30 g/907 kg (DM basis). Steers were fed ractopamine hydrochloride (Optaflexx 45, Elanco, Indianapolis, IN, USA) at a rate of 300 mg per steer for the final 28-d before harvest. A slick bunk management approach was used with feed bunks visually assessed for residual feed daily at 0700 daily [13]. Fresh feed was manufactured once per day at 0800 for each treatment in a single batch using a mixing wagon (2.35 m^3^; scale readability 0.454 kg) [12]. Orts were collected, weighed, and dried in a forced air oven at 100 °C for 24 h to determine DM content if carryover feed went out of condition or was present on weigh days.

Diets presented in Table 1 are actual diet DM formulation based upon weekly ingredient DM analyses (drying at 60 °C until no weight change) with weekly feed batching records [14] and tabular nutrient values for crude protein (CP), neutral detergent fiber (NDF), acid detergent fiber (ADF), ash, ether extract (EE) and tabular energy values according to [15].

**Table 1:** Actual diet formulation and tabular nutrient composition ^1,2^.

	DOF					
Ingredient Inclusion, %	1–21	22–40	41–82	83–115	116–178	179–209
Dry-Rolled Corn	60.85	31.74	23.64	20.38	16.52	48.20
Liquid supplement	6.08	6.15	5.90	6.20	5.11	4.94
DDGS	15.23	15.29	15.32	14.42	15.31	15.08
Oat Hay	-	-	-	15.08	-	-
Wheatlage	17.84	14.71	-	-	-	-
HMC	-	32.11	23.79	43.92	33.17	-
Corn Silage	-	-	31.35	-	29.90	31.78
**Diet Composition**						
Dry Matter,%	64.64	62.74	58.79	79.46	56.71	54.54
Crude Protein,%	13.53	13.50	12.83	13.33	12.95	12.82
Neutral Detergent Fiber,%	22.48	20.83	24.67	21.11	24.19	24.84
Acid Detergent Fiber,%	12.08	11.03	13.57	11.19	13.24	13.65
Ash,%	6.75	6.59	6.58	6.48	6.07	6.03
Ether Extract,%	3.61	3.61	3.60	3.59	8.19	8.03
NEm, Mcal/kg	2.01	2.05	2.01	2.05	2.00	1.97
NEg, Mcal/kg	1.32	1.36	1.35	1.36	1.35	1.32

^1^ All values except for DM on a DM basis. 2 Tabular NE and nutrient values from [15] and actual DM composition from weekly DM assays.

### 2.5. Growth Performance Calculations

All steers were weighed individually on d − 1, 1, 63, 112, 125, 153, 181, and 209. All interim period growth performance data was based upon live weight reduced 4% to account for digestive tract fill. Cumulative growth performance was based upon initial BW (average BW from d − 1 and 1 with a 4% shrink applied to account for digestive tract fill) and final BW from d 209 (FBW, shrunk 4%) and carcass-adjusted final BW (HCW divided by 0.625. Average daily gain (ADG) was calculated as the difference between FBW and initial shrunk BW, divided by days on feed and feed efficiency was calculated from ADG/DMI.

Growth performance was used to calculate performance-based dietary NE to determine efficiency of dietary NE utilization. The performance-based dietary NE was calculated from daily energy gain (EG; Mcal/d): EG = ADG^1.097^ × 0.0557W^0.75^, where W is the mean equivalent shrunk BW [kg; [16]] from mean feeding shrunk BW and final BW at 28% estimated empty body fatness (AFBW) was calculated as: [median feeding shrunk BW × (478/AFBW), kg;] [7,8]. Maintenance energy (EM) was calculated by the equation: EM = 0.077 × median feeding shrunk BW^0.75^. Dry matter intake is related to energy requirements and dietary NEm (Mcal/kg) according to the following equation: DMI = EG/(0.877 NEm − 0.41), and can be resolved for estimation of dietary NEm by means of the quadratic formula $x=\frac{-b\pm \sqrt{b^{2}-4ac}}{2c}$, where a = −0.41 EM, b = 0.877 EM + 0.41 DMI + EG, and c = −0.877 DMI [17]. Dietary NEg was derived from NEm using the following equation: NEg= 0.877 NEm − 0.41 [18]. The maintenance coefficient (MQ) was determined from DMI, EG and tabular energy values.

### 2.6. Hip Height Measurement

Hip height (HH) measurement was performed on all steers on d 1, 63, 112, 125, 153, 181, and 209. A tape measure was secured to the top of the chute 183 cm from the chute flooring. The tape measure was pulled down to meet the hip of the steer perpendicularly. This measurement was then subtracted from the height above the chute floor to calculate HH.

### 2.7. Urea Space Determination

Urea space measurements were determined using the technique described in [19] for sentinel steers (*n* = 1 steer/pen), selecting a steer that represented the median weigh of each pen. One steer per pen was used due to logistics of sample collection and time constraints required to ensure timely feeding of steers on collection days. The urea solution was 20% urea in 0.9% saline solution (*w*/*v*) and was infused at a rate of 0.75 mL solution per kilogram of shrunk body weight or 150 mg urea per kilogram of live body weight. Before infusion, the solution was filtered through a 0.8 μm filter unit. The urea solution was mixed and filtered within 24 h of infusion. The solution was stored at 4 °C. Neither feed nor water were withheld prior to infusions. Urea infusions were accomplished using jugular venipuncture with a 16-gauge × 1 ½ inch needle to ensure that all the infusate would go directly into the blood supply and reduce the possibility of injecting the infusion solution subcutaneously. The tubing was flushed with 5 mL of heparinized saline (100 units heparin per mL of saline). Before collecting a sample, approximately 5 mL of blood was drawn into a syringe and then reinjected through the catheter; thus, allowing for all blood samples collected to be fresh and have a homogeneous urea concentration. A 10 mL blood sample was collected (T0) before injecting any infusate into the steer. Blood samples were stored on ice and in a sterile Vacutainer^®^ (Becton Dickinson Vacutainer Systems, Franklin Lakes, NJ, USA) with no additive and centrifuged at 4 °C at 1250× *g* to harvest sera. The predetermined volume of infusate, 0.75 mL solution per kilogram of shrunk live body weight, was infused within 2 min using a 60 mL syringe. Infusion times were recorded on a data sheet, using the mid-point of infusion as the starting time, along with animal number, weight, and quantity of infusate injected. Twelve minutes later (T12), a blood sample was taken using the same collection procedure as above. The accuracy of the volume of infusate injected was gravimetrically determined by infusing into three volumetric flasks, once each at the beginning, mid-point, and end of the sampling day. Blood samples were centrifuged at 3000× *g* for 20 min. Serum urea nitrogen (SUN) analysis was performed within 24 h according to the methods described by [20].

Percent urea space (US) was calculated using the equation described by [21]: $\frac{mg\;urea\;infused}{Delta\;SUN\;\left(\frac{mg}{dL}\right)\times EBW)}$, where Delta SUN is the change in SUN concentration of the blood between T0 and T12 and EBW was calculated as unshrunk BW multiplied by 0.857. All samples were analyzed in triplicate and samples were considered for reruns if the coefficient of variation within triplicate runs was greater than 10%. The intra- and inter- assay coefficient of variation were less than 12%. Percent empty body water (EBH_2_O), percent empty body fat (EBF) and percent carcass protein of each steer was calculated with the following equations [22].

1. % EBH_2_O = 59.1 + 0.22 × US% − 0.04 × EBW
2. % EBF = 19.5 − 0.31 × US% + 0.05 × EBW
3. % Carcass Protein = 16.7 + 0.07 × US% + 0.01 × EBW

### 2.8. Carcass Trait Determination

Steers were marketed and harvested at a commercial abattoir when treatment blinded personnel determine that 60% of the population has sufficient fat cover to grade USDA Choice. Steers were loaded onto trucks, shipped 238 km, and harvested the following day at Tyson Fresh Meats in Dakota City, NE. Liver abscess prevalence and severity was determined by a trained technician using the Elanco system as: Normal (no abscesses), A− (1 or 2 small abscesses or abscess scars), A (2 to 4 well organized abscesses less than 2.54 cm diameter), or A+ (1 or more large active abscesses greater than 2.54 cm in diameter with inflammation of surrounding tissue) [23].Video image data was obtained from the plant for rib eye area, rib fat, kidney-pelvic-heart fat, calculated USDA Yield Grade [24] and USDA marbling scores. Dressing percentage was calculated as HCW/(final BW × 0.96). A covariate evaluation of ribeye size on hot carcass weight to evaluate ribeye area at a common weight was used. Estimated empty body fat (EBF) percentage and AFBW was calculated from observed carcass traits [25], and proportion of closely trimmed boneless retail cuts from carcass round, loin, rib, and chuck was determined according to the equation described by [26].

### 2.9. Management of Pulls and Removals

All steers that were pulled from their home pen for health evaluation were monitored in individual hospital pens prior to being returned to their home pens. When a steer was moved to a hospital pen the appropriate amount of feed from their home pen was removed and transferred to the hospital pen. If the steer in the hospital returned to their home pen, this feed remained credited to the home pen. If the steer did not return to their home pen, all feed that was delivered to the hospital pen was deducted from the feed intake record for that particular pen back to the date the steer was hospitalized. Three steers were removed from their home pens during this experiment for treatment and returned to their home pen upon recovery, with illness reasons unrelated to treatment.

### 2.10. Statistical Analysis

Growth performance, carcass traits, and efficiency of dietary NE utilization was analyzed as a randomized complete block design using the GLIMMIX procedure of SAS 9.4 (SAS Inst. Inc., Cary, NC, USA) with pen as the experimental unit. The model included the fixed effect of treatment and random effect of pen location. Least squares means were generated using the LSMEANS statement of SAS and treatment effects were analyzed using the pairwise comparisons PDIFF and LINES option of SAS 9.4. Distribution of USDA Yield and Quality grade data as well as liver abscess prevalence and severity were analyzed as a multinomial distribution in the GLIMMIX procedure of SAS 9.4 with fixed effect in the model as described previously. Regression coefficients for empty body percentages of water, fat and protein were calculated using PROC GLM. Linear and quadratic models for body composition parameters were compared using the adjusted r-squared value as the selection criteria. Regressions coefficients for the two treatment groups were compared using the procedures detailed in [27]. An α of 0.05 or less was used to determine significance with tendencies between 0.05 and 0.10.

## 3. Results

### 3.1. Growth Performance Day 1 to Day 112

Growth performance and carcass data from this experiment are located on Table 2. HIW steers had heavier (*p* = 0.01; 356 vs. 273 kg) initial BW compared to LIW by design, and HIW treatment remained heavier (*p* = 0.01; 527 vs. 442 kg) through d 112. Initial HH was greater (*p* = 0.01) for HIW steers and remained greater (*p* = 0.01) through d 112 compared to LIW. Daily HH change from study initiation until d 112 was greater for LIW steer (*p* = 0.03). ADG did not differ (*p* = 0.50; 1.66 vs. 1.61 kg) during this feeding period. DMI (kg) was greater for HIW steers (*p* = 0.01; 11 vs. 9.62 kg) compared to LIW. LIW steers were more efficient (G:F) (*p* = 0.01) from study initiation to d 112 than HIW. An increased maintenance coefficient (MQ) was noted for HIW (*p* = 0.02) compared to LIW steers. Observed dietary NEm and NEg (Mcal/kg) based on growth performance were greater for LIW (*p* = 0.02).

### 3.2. Growth Performance Day 113 to Day 209

After reimplantation on d 112, HH daily gain was not significantly different (*p* = 0.26) between treatment groups. No difference was noted for ADG (*p* = 0.26) during this period. DMI was greater for HIW steers (*p* = 0.01; 11.91 vs. 10.78 kg) compared to LIW steers. LIW steers tended (*p* = 0.08) to have better feed conversion efficiency (G:F). No difference between treatments was noticed for MQ (*p* = 0.85) or observed NEm or NEg (*p* = 0.99).

### 3.3. Cumulative Growth Performance

BW was increased for HIW cattle (*p* = 0.01; 701 vs. 610 kg) and had greater final HH (*p* = 0.01) compared to LIW steers. Carcass-adjusted (HCW/0.625) BW was greater for HIW steers (*p* = 0.01) and carcass adjusted ADG (*p* = 0.04) was also greater for HIW. Cumulative HH daily gain was greater for LIW steers (*p* = 0.04). Cumulative DMI was increased for HIW steers (*p* = 0.01; 11 vs. 9.61 kg). LIW steers were more efficient as measured on either a live (*p* = 0.01) or carcass adjusted (*p* = 0.01) basis. No differences were noted between treatment groups for cumulative live, or carcass adjusted MQ (*p* = 0.19 and *p* = 0.29, respectively). Observed dietary NEm and NEg (*p* = 0.17) did not differ between treatment groups when measured over the entire experiment.

### 3.4. Carcass Composition Determined from Urea Space

**Figure 1:**
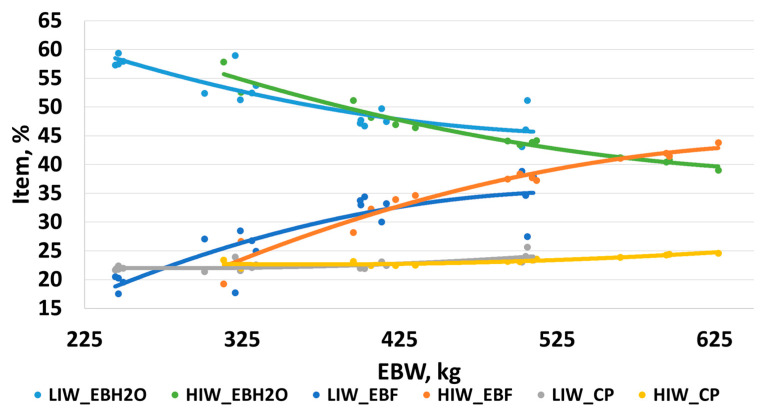
Treatments included (1) Light Initial Weight (LIW) and (2) Heavy Initial Weight (HIW). Empty body water (EBH2O), fat (EBF), and protein (CP) as determined by urea space dilution regressed against empty body weight (EBW). Empty body water, %: LIW = 0.0001EBW^2^ − 0.1445EBW + 86.17, R^2^ = 0.79; HIW = 0. 0.0001EBW^2^ − 0.1461EBW + 91.59, R^2^ = 0.96. Empty body fat, %: LIW = –0.0002EBW^2^ + 0.1972EBW − 18.65, R^2^ = 0.76; HIW = − 0. 0.0001EBW^2^ + 0.1995EBW –26.28, R^2^ = 0.95. Empty body protein, %: LIW = 0.00004EBW^2^ − 0.0232EBW + 25.31, R^2^ = 0.51; HIW = 0. 0.00003EBW^2^ − 0.0238EBW + 27.04, R^2^ = 0.80. Regression coefficients did not differ between LIW and HIW for urea space calculations of empty body water, fat, or protein (*p* ≥ 0.70).

Regression coefficients for empty body percentages of water, fat and protein were calculated using PROC GLM and represented on Figure 1. Coefficients for the two treatment groups were compared using procedures as detailed in [27]. Regression coefficients did not differ between LIW and HIW for urea space calculations of empty body water, fat, or protein (*p* ≥ 0.70). A quadratic response was noted for empty body fat (EBF), empty body water (EBH20), and carcass protein (CP). Empty body water decreased at an increasing rate as both treatment groups gained weight. Empty body fat increased at a decreasing rate as the treatment groups got heavier. Carcass protein gradually increased as the cattle put on weight. The quadratic equations for HIW had greater r-squared than LIW across the three studied components. With the HIW treatment group starting and ending the trial ~80 kg heavier, the proportion of each component (EBF, EBH20, and CP) would have been greater, resulting in a greater predictive outcome.

### 3.5. Carcass Characteristics

Carcass data from this experiment are located on Table 2. HIW steers had greater HCW (*p* = 0.01) compared to LIW cattle. No difference was detected for dressing percentage (*p* = 0.58) between treatment groups. A tendency was found (*p* = 0.06) for HIW steers to have a larger ribeye area (REA), but HCW at the same REA was lighter for LIW steers (*p* = 0.01), meaning they had greater muscularity at a lighter HCW. HIW steers tended (*p* = 0.09) to have more rib fat (RF). HIW steers had a greater degree of marbling (*p* = 0.05) and a greater numerical yield grade (YG) (*p* = 0.04). LIW steers had a greater proportion (*p* = 0.04) of YG1 carcasses compared to HIW steers. LIW steers had greater retail yield (*p* = 0.04) and empty body fat (EBF, %) (*p* = 0.02). Adjusted final body weight (AFBW, kg) was greater for HIW steers (*p* = 0.01; 627 vs. 573). Liver abscess severity did not differ between treatment groups (*p* = 0.53).

## 4. Discussion

Within this experiment, genetics across both treatment groups were similar as they were sourced from a singular ranch with alike gene pools. Both the LIW and HIW cattle were fed a common 16% roughage inclusion diet throughout the duration of the 209 days. Most other research conducted within this field have confounding factors of genetics and comparison of multiple diets when comparing cattle of different frame sizes. The growth performance results in this experiment combined with urea space composition results suggest that these cattle did not differ in frame size, but potentially differ in age as there was a 60-day calving window within the entire group. The HIW steers had increased ADG and final BW, but a poorer G:F compared to LIW cattle. This is similar to the results in calf-fed Holstein steers [2]. Additionally, [28,29] indicated that increased age at weaning resulted in similar performance differences to those noted in the present study. This suggests that composition of gain on HIW, as shown by the urea space determination and carcass results, had a greater proportion of adipose tissue as opposed to muscle later in the feeding period. It is evident the cattle were compositionally growing at the same rate, but with different starting intercepts, suggesting that the higher efficiency in the LIW cattle was due to composition of muscle gain for a longer period. Regression coefficients did not differ between LIW and HIW for urea space calculations of empty body water, fat, or protein (*p* ≥ 0.70). This shows that the rate of change when regressing EBF, EBW, and CP against EBW, there was no difference between treatment groups. Conventional knowledge may have predicted the LIW cattle to fatten faster with this treatment group starting on a 16% roughage diet at a lighter entry weight, however; under the conditions of this experiment, LIW did not get fatter faster or affect targeted composition when placed on a 16% roughage diet through the growing-finishing phase. This may suggest that even though the LIW cattle would take longer to reach the same compositional end point as the HIW steers, LIW treatment group grew at the same rate as HIW. However, in this current experiment, all cattle were harvested on the same date, meaning we can only speculate how the LIW cattle would have performed with additional days on feed.

Feedlot producers may do a pre-sort or an end-sort of cattle in their standard practice, but this research suggests that putting cattle with similar entry weights together may be beneficial to ensure steady growth rates to capitalize on an optimal carcass composition upon harvest. This method would help level the field of competition when referencing bunk space.

Work done at Cornell University [30] used Holstein and Angus bulls and heifers to study the relationship of full body weight, empty body weight, and shrunk body weight. In that experiment, SBW was an excellent predictor of EBW, and authors concluded that EBW could be used as a good predictor of warm carcass weight. Within our experiment, HIW steers had greater HCW and Yield Grade compared to LIW steers, but this information is predictable with this trial creating as stark of a difference as possible with cattle of similar genetic make.

## 5. Conclusions

Previous work from our lab [1] demonstrated that a single growing-finishing diet achieved a similar outcome compared to a two-diet system. The results of the current experiment show that the larger and smaller tails of a population of cattle grew at similar rates with similar rates of accretion for both protein and fat. Feeding smaller cattle a diet containing 84% concentrate at near ad libitum intake did not result in more rapid fat accumulation. This research suggests that there is no need to feed separate diets to optimize growth, but to sort cattle of like weight together and feed lighter weight cattle longer. The regression analysis supports that growth proceeds at the same rate but it began at different starting points because of initial weight. In conclusion, HIW steers had greater growth, but poorer feed efficiency compared to LIW steers. Steers with HIW produced fatter carcasses with a greater degree of marbling.

## Figures and Tables

**Table 2:** Growth performance for steers with light or heavy initial weights fed a common diet for 209 d.

	Placement BW: Light Initial Weight (LIW) and Heavy Initial Weight (HIW)			
Item	LIW	HIW	SEM	*p*-Value
No. pens	5	5	-	-
No. steers	35	35	-	-
Weaning weight ^1^, kg	217	291	2.5	0.01
Pre-conditioning BW (d − 1) ^2^, kg	283	367	1.6	0.01
Pre-conditioning ADG ^3^, kg	1.17	1.36	0.041	0.01
Hip Height (HH), cm				
Initial	114.45	122.32	0.188	0.01
d 112	127.15	133.40	0.116	0.01
d 209	135.38	140.51	0.291	0.01
HH daily gain, cm				
Initial to 112	0.1135	0.0988	0.00181	0.03
d 113 to 209	0.0848	0.0734	0.00346	0.26
Cumulative	0.1003	0.0871	0.00172	0.04
BW, kg				
Initial ^4,5^	273	356	1.6	0.01
d 112 ^5^	442	527	8.2	0.01
d 209 ^5^	610	702	5.6	0.01
Carcass-Adjusted (HCW/0.625)	626	723	10.3	0.01
Average daily gain (ADG), kg				
Initial to 112	1.51	1.53	0.070	0.50
d 113 to 209	1.73	1.80	0.107	0.26
Cumulative (live)	1.61	1.66	0.034	0.05
Cumulative (Carcass-adjusted)	1.69	1.76	0.048	0.04
Dry matter intake (DMI), kg				
d 1 to d 112	8.61	10.22	0.429	0.01
d 113 to d 209	10.78	11.91	0.469	0.01
Cumulative	9.62	11.00	0.381	0.01
ADG/DMI (G:F)				
Initial to d 112	0.175	0.150	0.0027	0.01
d 113 to d 209	0.161	0.151	0.0044	0.08
Cumulative (live)	0.168	0.151	0.0031	0.01
Cumulative (Carcass-adjusted)	0.176	0.160	0.0031	0.01
Maintenance coefficient ^6^, Mcal/BW^0.75^, kg				
Initial to 112	0.098	0.106	0.0022	0.02
d 113 to d 209	0.042	0.042	0.0030	0.85
Cumulative (live)	0.078	0.083	0.0028	0.16
Cumulative (Carcass-adjusted)	0.071	0.075	0.0023	0.22
Observed-to-expected (O/E) DMI				
Initial to 112	1.11	1.16	0.013	0.02
d 113 to d 209	0.83	0.83	0.012	1.00
Cumulative (live)	1.00	1.03	0.015	0.19
Cumulative (Carcass-adjusted)	0.97	0.99	0.013	0.29
Observed NEm, Mcal/kg				
Initial to 112	1.85	1.78	0.778	0.02
d 113 to d 209	2.33	2.33	1.429	0.99
Cumulative (live)	2.00	1.96	1.036	0.17
Cumulative (Carcass-adjusted)	2.05	2.03	0.975	0.29
Observed NEg, Mcal/kg				
Initial to 112	1.21	1.15	0.683	0.02
d 113 to d 209	1.64	1.64	1.254	0.99
Cumulative (live)	1.34	1.31	0.909	0.17
Cumulative (Carcass-adjusted)	1.39	1.37	0.854	0.29
O/E NEm				
Initial to 112	0.91	0.88	0.009	0.02
d 113 to d 209	1.17	1.17	0.017	1.00
Cumulative (live)	0.99	0.97	0.011	0.15
Cumulative (Carcass-adjusted)	1.02	1.01	0.011	0.43
O/E NEg				
Initial to 112	0.90	0.85	0.012	0.02
d 113 to d 209	1.22	1.22	0.020	0.93
Cumulative (live)	0.99	0.97	0.016	0.23
Cumulative (Carcass-adjusted)	1.03	1.02	0.013	0.29
HCW based growth (d 1 to 209)				
Initial HCW ^7^, kg	153.32	207.76	1.0	0.01
Final HCW, kg	391	452	6.4	0.01
HCW ADG, kg	1.14	1.17	0.030	0.09
HCW G:F	0.119	0.106	0.0020	0.01
Carcass traits				
HCW, kg	391	452	6.4	0.01
Dressing ^8^, %	64.22	64.45	0.398	0.58
REA, cm^2^	97.42	103.03	0.336	0.06
HCW at equal REA	395	449	4.1	0.01
RF, cm	1.52	1.75	0.049	0.09
Marbling ^9^	472	504	11.4	0.05
Yield Grade	2.94	3.41	0.193	0.04
Retail Yield	50.23	49.26	0.394	0.04
Estimated empty body fatness (EBF) ^10^, %	30.59	32.79	0.731	0.02
Fianl BW at 28% EBF ^10^, kg	573	627	24.1	0.01
HCW grouping, %				
Less than 363 kg	5.7	0.0	-	0.01
363 to 408 kg	71.4	5.7	-	-
408 to 454 kg	22.9	40.0	-	-
454 to 476 kg	0.0	42.9	-	-
Greater than 476 kg	0.0	11.4	-	-
Yield Grade, %				
1	5.7	2.9	-	0.04
2	48.6	31.4	-	-
3	40.0	42.8	-	-
4	5.7	20.0	-	-
5	0.0	2.9	-	-
Quality Grade, %				
Select	22.8	11.4	-	0.22
Choice	74.3	85.7	-	-
Prime	2.9	2.9	-	-
Liver Scores ^11^, %				
Normal	94.2	97.1	-	0.53
A-	2.9	2.9	-	-
A	0.0	0.0	-	-
A+	2.9	0.0	-	-

^1^ Average on 10/19 and 10/20/2021 BW, no shrink applied. ^2^ Body weight captured on 12/14/2021, no shrink applied. ^3^ Difference between pre-conditioning BW and weaning weight divided by 56 d. ^4^ Average of a 2 d BW collected on 12/14 and 12/15 was used as the initial on test BW. ^5^ Shrunk 4% to account for digestive tract fill. ^6^ Estimated maintenance requirements. ^7^ Initial HCW, kg = (0.2598 × initial shrunk BW, kg^1.1378^). ^8^ Calculated as: (HCW/final BW shrunk 4%) × 100. ^9^ 400 = small^00 10^ Calculated according to the equations described by [25]. ^11^ Determined according to the Elanco Liver Scoring System.

## Data Availability

Data available on request due to restrictions.
